# Type 1 segmental Darier disease unmasked by enfortumab vedotin

**DOI:** 10.1016/j.jdcr.2026.08.030

**Published:** 2026-08-28

**Authors:** Nina B. Curkovic, Yash Kumar, Sheba Jaison Joy, Tian Zhang, Travis Vandergriff, Meghan Heberton

**Affiliations:** a Department of Dermatology, University of Texas Southwestern Medical Center, Dallas, Texas; b Department of Dermatology, Case Western Reserve University School of Medicine, Cleveland, Ohio; c Department of Internal Medicine, University of Texas Southwestern Medical Center, Simmons Comprehensive Cancer Center, Dallas, Texas

**Keywords:** acantholysis, dyskeratosis, Enfortumab vedotin, mosaicism, segmental Darier disease, toxic erythema of chemotherapy

## Introduction

Darier disease (DD) is a rare autosomal dominant genodermatosis with mutations in the *ATP2A2* gene. Subsequent dysfunctional endoplasmic reticulum Ca^2+^ ATPase (SERCA2) protein leads to dysregulated intracellular calcium signaling within keratinocytes producing the characteristic histologic finding of acantholytic dyskeratosis.^1^ Clinically, DD presents in adolescence or early adulthood with red-brown keratotic papules in a seborrheic distribution. Nail changes, such as longitudinal erythronychia, leukonychia, or ridging are also frequently present. The disease follows a fluctuating, chronic course with triggers of acute flares including excess heat/sweating, UVB, friction, stress, and certain medications (eg diltiazem, lithium, interferon alpha 2a). Patients are also susceptible to developing bacterial and viral superinfections. Less common subtypes, including segmental DD, have been described and attributed to genetic mosaicism caused by postzygotic *ATP2A2* mutations. Segmental DD clinically demonstrates more variable foci of lesions, such as in type 1 segmental DD, where lesions follow a Blaschkoid distribution.^1^^,^^2^

Enfortumab vedotin (EV) is a nectin-4 directed antibody-drug conjugate that is used in advanced urothelial carcinoma. Expression of nectin-4, a cell adhesion molecule, in keratinocytes leads to a range of frequent, off target cutaneous reactions, including toxic erythema of chemotherapy and more severely, Stevens-Johnson syndrome/toxic epidermal necrolysis or Stevens-Johnson syndrome/toxic epidermal necrolysis-like toxic erythema of chemotherapy.^3^^,^^4^ Herein, we present a case of EV unmasking type 1 segmental DD in a patient being treated for metastatic bladder cancer.

## Case report

A 54-year-old male undergoing treatment for metastatic bladder cancer presented to the dermatology clinic with a pruritic eruption on the right chest. The patient reported onset of the eruption approximately 1 week after receiving the third treatment of a clinical trial regimen with EV and an anti-CD47 agent. He reported a similar, recurrent rash involving his right shoulder during his time in the military that intermittently flared and remitted. His prior oncologic treatment history included six cycles of chemotherapy with methotrexate-vinblastine-doxorubicin-cisplatin, followed by cystectomy, and pembrolizumab for recurrent peritoneal metastases. Of note, he reported a similar eruption on the right chest after completing methotrexate-vinblastine-doxorubicin-cisplatin, which self-resolved without treatment.

Given history of herpes zoster and concern for recurrence, the referring oncologist initiated therapeutic valacyclovir prior to dermatologic evaluation with mild improvement. Upon evaluation, the patient denied any new exposures but endorsed frequent exercise, including outdoor workouts in significant heat. He denied any family history of similar eruptions.

Physical examination demonstrated eroded papules and pseudovesicular plaques, distributed in a Blaschkoid pattern involving the right back, right shoulder, right arm, and right chest (Figs 1 and 2). Scattered eroded papules were also present on the left chest and back. Initial differential diagnosis included segmental DD versus less likely contact dermatitis, interface dermatitis related to EV, and disseminated herpes zoster. A Varicella zoster virus polymerase chain reaction obtained from lesional skin was negative, and valacyclovir was subsequently discontinued. Two punch biopsies for hematoxylin and eosin stain were obtained from the right chest and right upper arm. Histopathology of both sites revealed focal acantholysis and dyskeratosis, consistent with DD (Fig 3).

**Fig 1:**
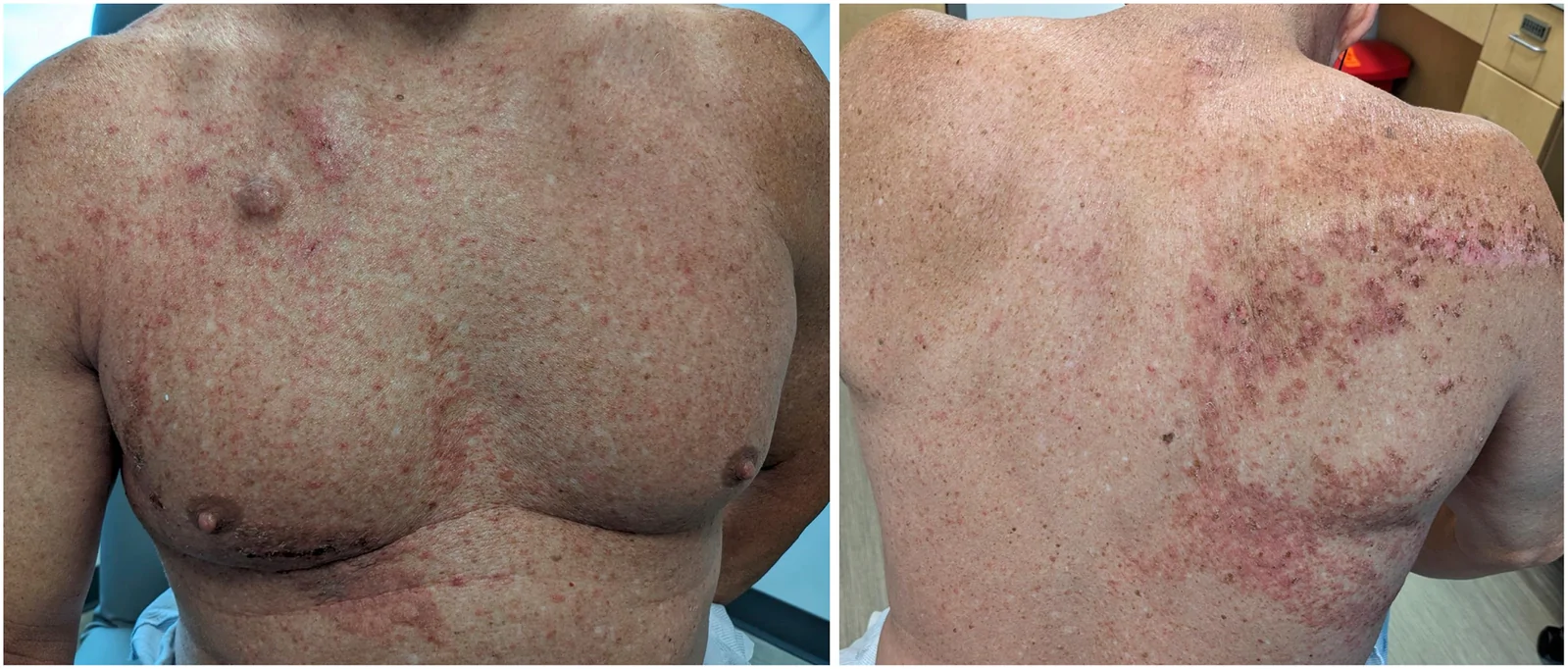
Cutaneous findings demonstrating red-brown eroded papules and pseudovesicular plaques involving the right back and right chest at presentation.

**Fig 2:**
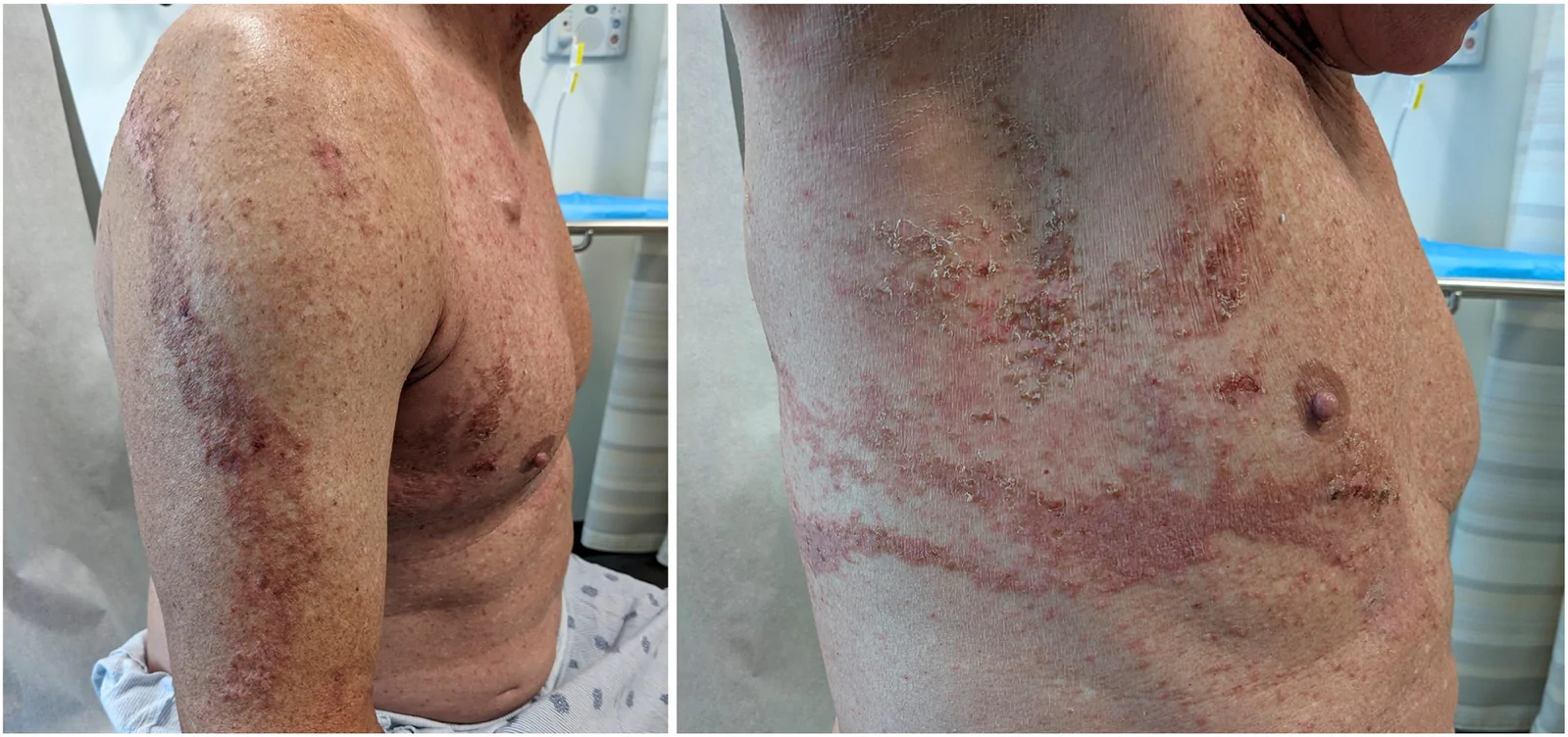
Cutaneous findings demonstrating red-brown eroded papules and pseudovesicular plaques involving the right shoulder, right arm, and right chest at presentation.

**Fig 3:**
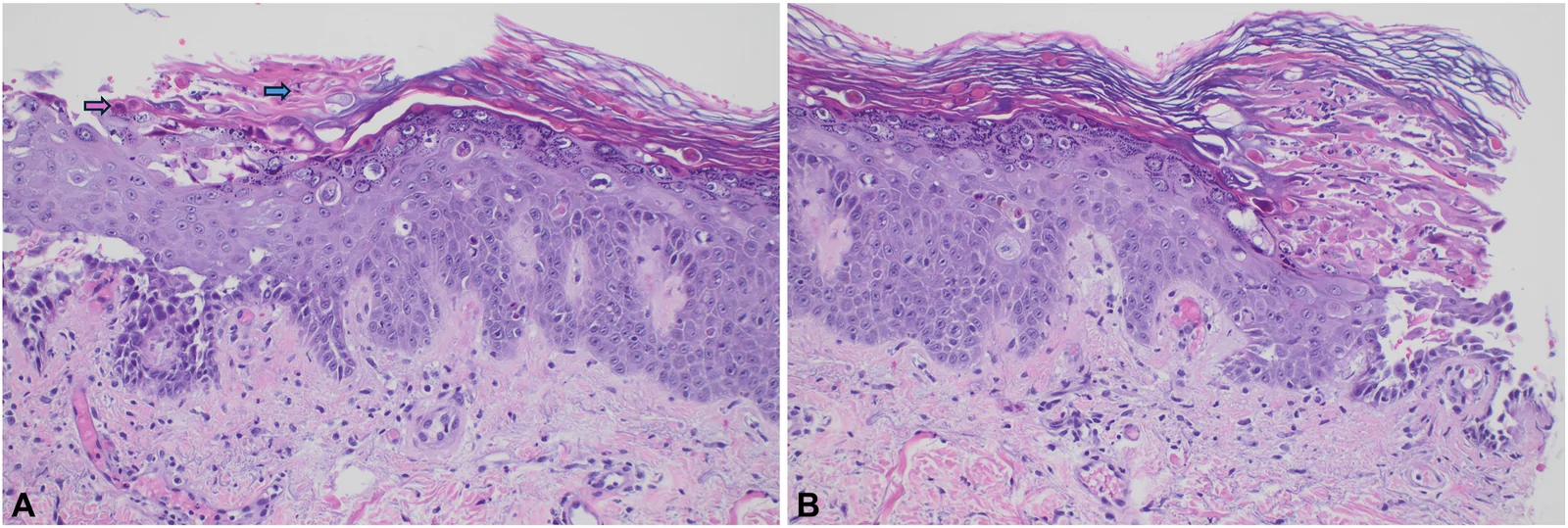
Skin histopathology demonstrating **(A)** acantholysis above the basal and spinous layers along with dyskeratosis demonstrating corps ronds (*pink arrow*) and grains (*blue arrow*) and **(B)** prominent acantholytic dyskeratosis on the right side of the frame with epidermal dysmaturation and occasional mitotic figures on the left side of the frame. Hematoxylin and eosin stain (H&E).

Overall, the patient’s history, physical examination, and histopathologic findings were most consistent with pre-existing type 1 segmental DD, likely unmasked following initiation of EV. To our knowledge, this is the first report of DD flare in association with EV. The patient was treated with topical triamcinolone and advised to avoid heat exposure and saw subsequent clinical improvement. EV and anti-CD47 study treatment were continued without interruption.

## Discussion

DD is a rare genodermatosis involving pathogenic variants in *ATP2A2*, infrequently presenting in a Blaschkoid distribution secondary to genetic mosaicism. Two variants of segmental DD have been described: type 1 and type 2 segmental DD. Type 1 segmental DD presents with lesions in a Blaschkoid distribution on a background of normal skin, while type 2 segmental DD presents with focal areas of varying severity on a background of involved skin.^2^ Clinically, our patient’s presentation with unilateral eroded papules and pseudovesicular plaques could mimic a zoster eruption. However, the history of an intermittently recurrent rash that crossed multiple dermatomes on a background of normal skin favored type 1 segmental DD. Histopathology demonstrating focal acantholysis and dyskeratosis, as well as negative viral polymerase chain reaction, further confirmed a suspicion of DD in this case.

EV is increasingly being utilized as a cancer therapy and has been recognized as a frequent cause of cutaneous reactions with variable morphologies and onset. Drug-related cutaneous events often occur early within the first cycle, with a range of 7 to 63 days after first infusion in one series.^5^ Though our patient’s eruption began approximately 1 week after a subsequent exposure to EV and could be consistent with previously reported EV-related events, it is important to note that not all dermatologic eruptions in patients undergoing treatment with EV should be classified as a drug eruption and may reflect unmasking of an underlying condition.

EV’s specificity for nectin-4 expressed in keratinocytes and adnexal epithelium has been proposed as a contributor to its dermatologic toxicity.^6^ As a result, it is possible that EV-related keratinocyte disruption may lower the threshold for clinical expression of DD, amplifying the impact of other potential exacerbating factors. For example, in this case, the patient’s history of exercise in heat may have further promoted epidermal barrier disruption and keratinocyte stress, accelerating a flare. Furthermore, subtle histopathologic EV-associated changes in this case, including epidermal dysmaturation and mitotic figures, highlighted a background of EV-related keratinocyte disruption amongst striking histopathologic findings of DD.^7^ Given symptoms of pruritis and localized disease, this patient was treated with topical steroids, though in refractory or more widespread disease, topical or systemic retinoids may be considered.

Several limitations preclude a clear causal relationship of EV in this patient’s cutaneous findings, including concurrent systemic oncologic therapy with an anti-CD47 agent, intermittently active disease history at baseline, and confounding by other risk factors for DD exacerbation. Despite this, the close temporal relationship to EV exposure and role of nectin-4 in cell adhesion support EV as a plausible precipitating factor for unmasking the patient’s DD. Unlike EV, anti-CD-47 agents have not been associated with significant cutaneous toxicity.^8^

This case report highlights the need for further study of EV’s impact on keratinocyte integrity and signaling, particularly in patients with underlying epidermal adhesion disorders. Early collaboration between dermatology and oncology is vital to establish a diagnosis, mitigate cutaneous toxicity, and minimize disruptions to therapy when cutaneous findings may not reflect direct adverse effects of therapy.

## Conflicts of interest

Dr Heberton is a consultant for Astellas. Additional nonrelevant disclosures include being an investigator for Lutris, a consultant and on the ad board for Blueprint Medicine and Revolutions medicine (Revmed). Dr Zhang received research funding from Merck, Janssen, Astra Zeneca, Pfizer, Astellas, Eli Lilly, Tempus, ALX Oncology, Janux Therapeutics, OncoC4, Exelixis, Bayer, Kura Oncology, Arcus Biosciences, and Daiichi Sankyo; they are on the consulting/advisory board of Merck, Exelixis, Sanofi-Aventis, Janssen, Astra Zeneca, Pfizer, Amgen, BMS, Eisai, Aveo, Eli Lilly, Bayer, Gilead, Novartis, EMD Serono, Dendreon, Xencor, Summit Therapeutics, Arcus Biosciences, MJH Associates, Vaniam, Aptitude Health, PeerView, Dava Oncology, and eChinaHealth. Drs Curkovic, Vandergriff and Authors Kumar and Joy have no conflicts of interest to disclose.
